# Effects of Photobiomodulation Therapy (PBMT) in the Management of Postoperative Pain After Third Lower Molar Extraction: A Narrative Review

**DOI:** 10.3390/jcm14155210

**Published:** 2025-07-23

**Authors:** Leopoldo Mauriello, Alessandro Cuozzo, Vitolante Pezzella, Vincenzo Iorio-Siciliano, Gaetano Isola, Gianrico Spagnuolo, Luca Ramaglia, Andrea Blasi

**Affiliations:** 1 School of Dental Medicine, University of Naples Federico II, Via S. Pansini 5, 80131 Naples, Italy; leopmau96@gmail.com (L.M.); alessandro.cuozzo@unina.it (A.C.); enzois@libero.it (V.I.-S.); gspagnuo@unina.it (G.S.); luca.ramaglia@unina.it (L.R.); andrea.blasi@unina.it (A.B.); 2 Unit of Periodontology, Department of General Surgery and Medical-Surgical Specialties, University of Catania, 95124 Catania, Italy; gaetano.isola@unict.it; 3 Therapeutic Dentistry Department, Institute for Dentistry, Sechenov University, Moscow 119991, Russia

**Keywords:** third lower molar, photobiomodulation therapy, postoperative pain, low-level laser therapy, oral surgery

## Abstract

**Background:** Third lower molar (TLM) extraction is one of the most common oral surgical procedures, often accompanied by postoperative pain and inflammation. In order to treat postoperative pain, different methods are used, mainly based on painkillers. PBMT may represent an adjunct to pain management. **Objective:** This narrative review aims to evaluate the efficacy of PBMT in reducing postoperative pain following TLM extraction. **Methods:** A comprehensive search was conducted to identify studies examining the use of PBMT for postoperative pain relief after TLM extraction. Four randomized controlled trials (RCTs) met the inclusion criteria and were analyzed qualitatively. **Results:** Two studies showed statistically significant reductions in pain with PBMT. Kahraman et al. reported lower pain scores in the intraoral PBMT (*p* = 0.001), with up to a 3.2-point reduction on the Visual Analog Scale (VAS). De Paula et al. found improved pain control using a dual-wavelength (808 + 660 nm) versus a single wavelength protocol (*p* = 0.031). The remaining studies showed non-significant results toward pain reduction. **Conclusions:** PBMT shows encouraging results in managing postoperative pain after TLM extraction, specifically with intraoral and multi-wavelength protocols. However, further studies are necessary to confirm its clinical utility.

## 1. Introduction

Third lower molar (TLM) extraction is one of the most common oral surgical procedures in dentistry [1]. TLMs often present with total or partial inclusion, a condition observed in 73% of young adults in Europe [2]. This is generally attributed to a discrepancy between tooth eruption and mandibular development [3]. Such anatomical discrepancies increase the risk of complications like pericoronitis, distal caries of the mandibular second molar (M2M), and periodontal issues, which often require surgical intervention [4]. Preoperative planning must consider various factors such as the proximity to the alveolar inferior nerve (AIN), bone resistance, and position of the lingual nerve, prompting the use of classification systems like Pell and Gregory, Winter, and the Pederson Index (e.g., Pell and Gregory; Winter; Pederson Index) [5].

After oral surgery, several complications may occur (e.g., AIN paresthesia, infection, alveolitis), but the most common is pain [1,6,7].

Pain is generally reported as the most frequent and distressing postoperative reason for discomfort following TLM extraction.

Standard treatment typically involves the prescription of paracetamol or non-steroidal anti-inflammatory drugs (NSAIDs), such as ibuprofen, with the occasional use of corticosteroids. While these pharmacological medications are generally effective, they are not without side effects [6].

Painkillers are the preferred choice to control postoperative pain. In particular, ibuprofen showed a better outcome in terms of pain relief compared to paracetamol [8]. In some cases, selective COX-2 inhibitors such as celecoxib are preferred for their combined effect on pain, swelling, and mouth opening [6], while corticosteroids did not show any statistically significant results for pain relief in both short- and long-term evaluations compared to placebo [9].

Photobiomodulation therapy (PBMT) has been proposed for several applications in oral surgery and dentistry. A systematic review indicated that PBMT may improve bone density and upregulate osteogenic markers such as Ocn and Runx2 after extractions, although heterogeneity in laser parameters limits direct comparisons [10]. However, one of the key issues of the several analyzed studies is the high variability of laser parameters, preventing the direct comparison and establishment of the optimal parameters for bone healing [10]. Photobiomodulation therapy has also proven to be significantly effective in reducing pain after mandibular TLM extraction. Patients who received PBMT showed a significant decrease in pain levels compared to simulated therapy. Specifically, the reduction was significant on all postoperative days assessed, including days 2 and 7, in which postoperative discomfort is at its peak [11].

Two recent systematic reviews further support PBMT’s efficacy. De Barros et al. found a mean reduction in pain scores on day 7 (MD −0.76, 95% CI −1.21 to −0.32) across 15 RCTs [12], while Lacerda-Santos et al. reported a standardized mean difference of −1.09 on day 3 (95% CI −1.63 to −0.55) in their meta-analysis of 33 RCTs [13]. These findings underscore PBMT’s growing role as a non-pharmacologic option for pain management after TLM extractions.

Additionally, in a recent prospective study, diode laser applied immediately after surgery showed significantly lower pain scores compared to the control group [14]. Hence, the main aim of the review is to evaluate the efficacy of laser therapy in the management of postoperative pain after TLM surgical extraction.

### 1.1. Low-Level Laser Therapy (LLLT) on Tissues and Safety

Light and laser emission is widely used in medicine. There are several types of lasers (e.g., CO_2_, Nd:YAG, Er:YAG, etc.), and the rationale behind their use is based on the biological tissue effects. Laser beams may be absorbed, reflected, scattered, or transmitted depending on the tissue composition and wavelength. Different wavelengths produce different outcomes on tissues: some penetrate deeply (e.g., Nd:YAG), while others primarily affect the surface (e.g., CO_2_ lasers) [15]. Low-power lasers can promote healing and tissue regeneration and therefore can be used in therapeutic applications like pain relief and wound healing [16]. Several studies reported positive effects of LLLT on fibroblast and collagen growth and cell regeneration in wound healing [17]. Tissue effects also include photothermal (heat-based), photoablative (removal of tissue without thermal damage), and photomechanical (shockwave-induced) effects [15]. Lasers are able to provide precise and efficient tissue interaction, making them a reliable tool in surgery and medicine. Nevertheless, the proper selection of wavelength and laser parameters is mandatory in order to achieve the best results with minimal adverse effects [15]. Laser devices should be carefully used in order to guarantee both user and patient safety. The potential risks of laser exposure may include eye injuries, burns, and toxic volatile compounds that could be inhaled. Therefore, eye protection is needed since the retina is particularly vulnerable to laser exposure, requiring protective eyewear; surgical and endotracheal tubes can ignite, requiring fire prevention measures to conclude that lasers can be used but with the necessary protections [18]. Finally, laser therapy also has its contraindications; it is, in fact, not suitable if the patient is affected by a malignant neoplasm or epilepsy or is pregnant [19].

Furthermore, laser therapy should be used with caution in patients with cardiac pacemakers due to potential electromagnetic interference (EMI) from laser power systems; however, in vitro data show that, while most lasers fall below the EMI safety thresholds, certain types, such as CO_2_ and ruby lasers, can slightly exceed the limits during standby, so maintaining a small distance from the implant is recommended [20]. Relative contraindications instead are blood diseases, local infection, chance of irradiating the gonads, photosensitive skin, or use of drugs that cause photosensitivity [19].

### 1.2. Low-Level Laser Therapy (LLLT) Effects on Pain

The first reaction to LLLT stimulation is local, but its effects are also systemic due to the body-wide transport of LLLT-mediated photoproducts in the blood and lymphatic system. In fact, it is believed that these photoproducts can influence both endorphins and prostaglandins such as IL-6, MCP-1, IL-10, and TNF-α in a dose-dependent manner for several days, hence justifying the anti-inflammatory, analgesic, regenerative, and desensitizing effects [17,21]. The pain modulation effects of LLLT after tooth extraction may be due to faster vascularization and, as a consequence, the faster formation of trabecular osteoid tissue [17]. Furthermore, the application of LLLT seems to show analgesic effects thanks to the increase in endogenous endorphins (β-endorphin) and the decrease in the activity of both C-fibers and bradykinin. Morphological changes were also observed in neurons, reducing the mitochondrial membrane potential and leading to neural conduction blockage. Interestingly, the anti-inflammatory effect of the LLLT could also be due to an increase in the number and diameter of lymphatic vessels and a lower permeability of blood vessels, resulting in edema reduction [21]. The clinical effects of LLLT may also be due to the expression of anti-apoptosis and pro-survival genes responsive to nuclear factor kappa B (NF-kB,) which could be triggered by specific laser wavelengths [22]. Furthermore, it seems that the application points may play a role in the effects of LLLT therapy on pain relief. In fact, after third lower molar extraction, Landucci et al. evaluated not only the effects of LLLT on pain, swelling, and trismus but also the application of the laser device, identifying six extraoral (along the masseter) and four intraoral points, showing a reduction in pain level in the test group [22]. Similarly, Escobar et al. applied a laser on six extraoral points (three points extending from the tragus to the outer corner of the mouth and the other three from the earlobe to the soft tissue pogonion) and one intraoral point, showing a statistically significant pain reduction in the test group.

## 2. Materials and Methods

An electronic search was conducted on PubMed, Web of Science, and Google Scholar. The following search strategy was used: ((“third”[All Fields] OR “thirds”[All Fields]) AND (“lower”[All Fields] OR “lowered”[All Fields] OR “lowering”[All Fields] OR “lowerings”[All Fields] OR “lowers”[All Fields]) AND (“molar”[MeSH Terms] OR “molar”[All Fields] OR “molars”[All Fields] OR “molar s”[All Fields]) AND (“extract”[All Fields] OR “extract s”[All Fields] OR “extractabilities”[All Fields] OR “extractability”[All Fields] OR “extractable”[All Fields] OR “extractables”[All Fields] OR “extractant”[All Fields] OR “extractants”[All Fields] OR “extracted”[All Fields] OR “extractibility”[All Fields] OR “extractible”[All Fields] OR “extracting”[All Fields] OR “extraction”[All Fields] OR “extractions”[All Fields] OR “extractive”[All Fields] OR “extractives”[All Fields] OR “extracts”[All Fields]) AND (“healed”[All Fields] OR “wound healing”[MeSH Terms] OR (“wound”[All Fields] AND “healing”[All Fields]) OR “wound healing”[All Fields] OR “healing”[All Fields] OR “healings”[All Fields] OR “heals”[All Fields]) AND (“low”[All Fields] AND (“level”[All Fields] OR “levels”[All Fields]) AND (“laser s”[All Fields] OR “lasers”[MeSH Terms] OR “lasers”[All Fields] OR “laser”[All Fields] OR “lasered”[All Fields] OR “lasering”[All Fields])) AND (“pain”[MeSH Terms] OR “pain”[All Fields])) AND ((randomizedcontrolledtrial[Filter]) AND (humans[Filter]) AND (english[Filter])).

**Figure 1:**
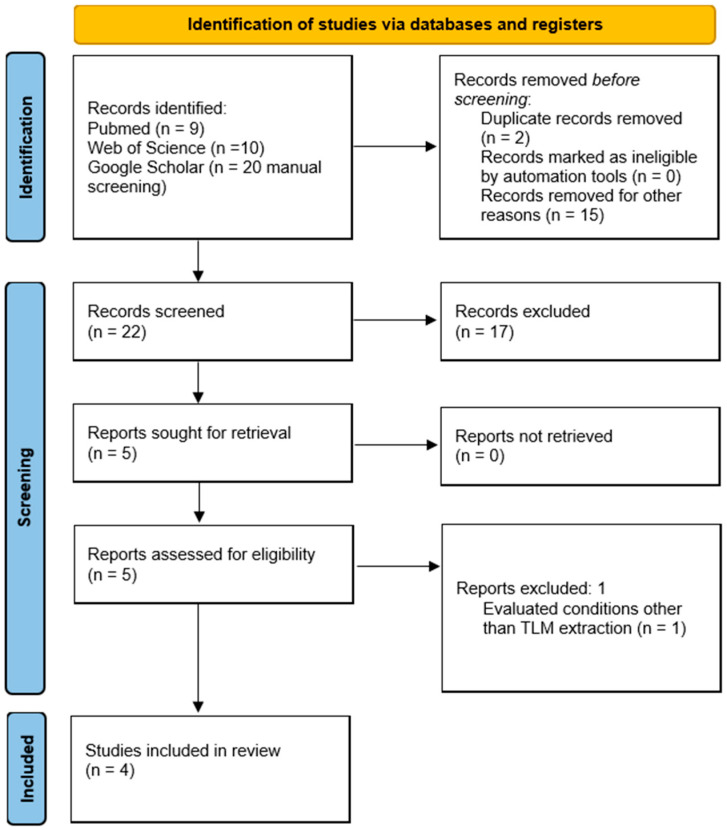
Flowchart article retrieval process.

The literature search was conducted using a systematic approach following the PRISMA 2020 framework to enhance transparency. Electronic searches were performed in PubMed, Web of Science, and Google Scholar. A detailed search strategy, including Boolean operators and RCT filters, was applied to PubMed and Web of Science. Due to limitations in advanced filtering, Google Scholar results were manually screened for relevance, language, study design, and duplicates. A PRISMA flow diagram has been included to illustrate the identification, screening, eligibility, and inclusion process (Figure 1). No formal risk-of-bias assessment was performed due to the narrative nature of the review.

The following studies were included in the literature review in accordance with the following inclusion criteria:

- Studies focused on the clinical effects of PBMT in pain reduction after TLM extraction;
- Studies performed in vivo on humans;
- Randomized controlled trials (RCTs).

The following studies were excluded from the review in accordance with the following exclusion criteria:

- Studies that use lasers to treat other oral conditions;
- Studies not available in English.

Five studies were found, and only one was excluded after title reading. Finally, four articles were selected. However, due to the scope and extent of this search, a wide-ranging comprehensive narrative review of PBMT on pain after third lower molar extraction was performed rather than a systematic review.

## 3. Results

Different levels of efficacy in postoperative pain reduction after TLM extractions using PBMT were recorded by two of the four selected randomized controlled trials (RCTs). Generally, the application of PBMT seems to positively affect patient-reported pain levels, but both the consistency and evaluation of the reported effect depend on several parameters.

Two of the studies (Fernando et al. and Pereira et al. [23,24]) reported no statistically significant differences in pain perception between PBMT-treated and placebo groups during follow-up. Despite the lack of statistical significance, both studies recorded a reduction in the PBMT groups, highlighting a potential analgesic effect that could have been affected by the small sample sizes.

On the other side, Kahraman et al. [25] found a statistically significant reduction (*p* = 0.001) in postoperative pain when PBMT was applied intraorally compared to both placebo and extraoral application groups. The intraoral group exhibited not only lower pain scores but also reported improvements in trismus and swelling, suggesting that laser application to the surgical site may enhance clinical efficacy, showing greater biostimulation and more targeted anti-inflammatory effects.

De Paula et al. provided additional insights by comparing two different PBMT wavelength combinations: 808 nm alone versus a dual-wavelength protocol (808 nm + 606 nm) [26]. The dual-wavelength approach recorded better pain control (*p* = 0.031), but since both groups received active PBMT, there could be evidence of synergistic effects of combined wavelengths with distinct tissue penetration profiles.

Across all the studies, diode lasers were the most used kind of laser, while the laser parameters such as energy density, treatment duration, and frequency differed significantly. Pain assessment was mostly performed using the Visual Analog Scale (VAS) on postoperative days 1, 3, and 7.

**Figure 2:**
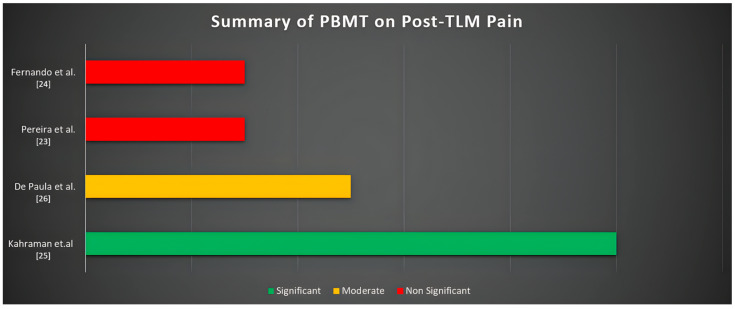
Significancy of the analyzed studies related to Post-TLM pain [23,24,25,26].

Finally, while pain was the primary endpoint, some studies also monitored secondary outcomes such as wound healing, edema, and trismus. In some cases, PBMT demonstrated positive effects in the treatment of all the secondary mentioned outcome conditions, even when the differences in pain levels were not statistically significant. The results are summarized in Table 1 and Figure 2.

## 4. Discussion

PBMT is a non-invasive photobiomodulation technique used in various medical fields for its potential to modulate inflammation, relieve pain, and promote tissue healing. The benefits of PBMT were assessed in reducing postoperative pain or accelerating socket healing following tooth extraction in healthy adults [27]. It is important to notice that all the analyzed studies focused their attention on the surgical extractions of third lower molars, which, excluding complicated cases, are generally associated with moderate pain that resolves spontaneously in a few days; therefore, the PBMT potential may be hidden or underestimated by the natural healing processes of the human body. However, LLLT can also reduce inflammatory markers and accelerate tissue remodeling, all of which contribute to better postoperative outcomes; therefore, it would be interesting to better focus the attention on patients with diabetes, cancer, or immunosuppression [27].

Therefore, the use of PBMT after TLM extraction in pain modulation seems to show promising results, but different degrees of efficacy were reported. The analyzed studies, however, are difficult to compare since different laser parameters and application techniques were used. A recent systematic review by Giansiracusa et al. (2024), which included 18 clinical studies, similarly found that PBMT significantly reduced postoperative pain and enhanced wound healing; however, the authors highlighted the need for standardized parameters due to heterogeneity in protocols and outcomes [28]. In fact, the laser wavelengths selected differ for penetration depths and biological effects. Red lasers show their effects more superficially and benefit mucosal and epithelial tissues, while infrared lasers can penetrate deeper and may affect connective tissue and bone [15,27]. The mechanism for the analgesic effects of PBMT is multifactorial; it is, in fact, able to determine a more stable conformation of the lipid bilayers, stabilizing nerve cell membranes [29]. Additionally, it seems to increase adenosine triphosphate production, enhance the redox systems of the cells, and therefore decrease pain transmission also thanks to the reduction in inflammation and biochemical markers such as IL-6, MCP-1, and TNF-α [21]. It seems that different protocols with different wavelengths may produce different effects, suggesting that intraoral application may determine better results [26]. Kahraman et al. found a significant difference in pain reduction with intraoral LLLT compared to transcutaneous application and placebo groups, supporting the above-cited statement [17]. Furthermore, the wavelength and dosage of PBMT appear to be crucial to the clinical results; in fact, all the included studies used different kinds of diode lasers, and their protocols varied in terms of energy density, exposure time, and treatment frequency. The better clinical results were obtained with a GaAIA laser with a wavelength of 800 nm. This is supported by Landucci et al., who demonstrated that a single dose of 7.5 J/cm^2^ at 10 mW, with an infrared wavelength of 780 nm, promotes pain relief and reduces trismus and edema because this wavelength penetrates deep into tissues [22,30]. Pereira et al. did not find any statistically significant results according to pain reduction; however, an improvement in edema and mucosal healing was observed. This is in line with a recent systematic review where no effects were observed in pain reduction [31]; however, the results may be due to the small sample size of only 20 patients. In contrast, a 2021 meta-analysis of 10 RCTs reported moderate but statistically significant reductions in pain and swelling following PBMT (SMD −0.53 and −0.60, respectively), although the impact on trismus was not significant, reinforcing the variability across outcomes [32]. The different protocols used highlight the need for a standardized approach to assure both the reproducibility and reliability of LLLT in clinical practice. Furthermore, pain is defined as an “unpleasant sensory and emotional experience associated with actual or potential tissue damage, or described in terms of such damage” [33]. Therefore, the emotional factor must be taken into account. Pain assessment tools, such as the visual analog scale (VAS), provide valuable insights, yet they remain subject to the patient’s perception, suggesting that a standardized method for pain evaluation should be found and used. This must be considered a limitation of the study. The short-term follow-up of the analyzed studies (3–7 days) is another limitation, although partial, since generally, the duration of pain after TLM extraction and tooth extraction ranges from 3 to 6 days after surgical intervention [34]. Additionally, while PBMT shows potential as a non-invasive and drug-free approach to pain management, its clinical results may be conditioned by the operator’s expertise in the use and application of the device. Lastly, the safety and contraindications of PBMT should be emphasized. While PBMT is generally considered safe, improper use can lead to adverse effects, such as tissue overheating or unintended nerve stimulation.

## 5. Conclusions

The reviewed studies suggest that PBMT could be an alternative to reduce postoperative pain, contributing to enhanced recovery. PBMT shows potential but requires further standardized, high-quality RCTs to establish its clinical role. Furthermore, one of the limitations of this narrative review is that a limited number of studies were included and a risk–bias assessment was not performed due to the nature of the study. Therefore, it is mandatory to find standardized protocols focusing on defining both kinds of lasers together with the wavelength and time of exposure in order to guarantee their clinical efficacy.

## Figures and Tables

**Table 1:** Characteristics and clinical findings of the analyzed studies.

Authors	Study Design	Setting	N° of Patients	Gender (Male/ Female)	Mean Age (Years)	Type of Laser Used	Application	Treatment	Pain Scale Used and Results	Time Evaluation (Days)
Fernando et al. (1993) [24]	RCT	University	64	N.A.	From 18 to 50	CBM Master 3 semiconductor laser of 830 nm wavelength and 30 mW mean power of beam	The laser was inserted into the post-extraction socket and used for 132 s for each extracted tooth	Test Group: third lower molar extraction plus laser application Control group: third lower molar extraction plus placebo	A verbal digital pain scale described by Giles (1984). No statistically significant difference was found at 1, 3, and 7 days. (*p* = 0.174; 0.772.; 0.884)	1 to 7 days after surgery
Kahraman et al. (2017) [25]	RCT	University	60	24M/36F	21.88 ± 4.32	Lumenis (Lumenis, Inc., Santa Clara, CA, USA) gallium aluminum arsenide (GaAlAs) 830 nm diode lasers set at 100 mW for extraoral Using and BTL 2000 (Medictinedic, Denmark) GaAlAs 830 nm diode lasers set at 100 mW for intraoral use	The laser was administered immediately before and after the extraction procedure. The applications lasted for 15 s. (3 J/cm^2^ of energy, 10.0 Hz, 63 mW) in continuous mode	Test Group 1: third lower molar extraction plus laser transcutaneous application Test Group 2: third lower molar extraction plus laser intraoral application Control group: third lower molar extraction plus placebo	VAS (visual analogic scale) was used. A statistically significant result was recorded between the intraoral laser application and the placebo group (*p* = 0.001) between the intraoral and the transcutaneous group (*p* = 0.001)	1 to 7 days after surgery
Pereira et al. (2022) [23]	RCT	University	20	N.A.	Over 18 years old	The GaAlAs laser (TheraLase, λ 660 nm and λ 808 nm 100 mW, ϕ ∼ 0.600 μm, tip divergence = 0.37 rad, CW, spot area 0.0283 cm^2^, DMC Equipamentos, São Carlos, SP, Brazil)	Two irradiations were performed intraorally in the middle of the extraction socket, at the buccal and lingual sides in contact with the alveolar sockets. The laser was irradiated immediately after tooth extraction and 3 and 7 days after surgery for 40 s in each session.	Test Group: third lower molar extraction plus laser application Control group: third lower molar extraction	VAS (visual analogic scale) was used. No statistically significant differences were found regarding the pain felt among the test and control groups	After tooth extraction and 3 and 7 days after surgery for 40 s in each session
De Paula et al. (2024) [26]	RCT	University	31	11M/20F	21 years (minimum 16; maximum 28)	Diode laser device (Therapy ECDMC Equipment, São Paulo, Brazil) used with a power of 100 milliwatts (mW) in continuous mode	The irradiation of dental sockets in the 808 (infrared laser) group was applied at three intraoral points with 2 Joules (J), for 20 s for each point. The dental sockets in the 808 + 606 (infrared + red laser) were irradiated in the same way.	Test Group: third lower molar extraction plus laser application with a wavelength of 808 nm Control group: third lower molar extraction plus laser application with a wavelength of 808 + 606 nm	VAS (visual analogic scale) was used. The pain assessment on the 7th day showed a statistically significant difference, being higher in the 808 + 660 group (*p* = 0.031).	After tooth extraction and 3 and 7 days after surgery for 40 s in each session

mW: milliwatt; nm: nanometers; μm: micrometers; VAS: visual analogic scale.

## Data Availability

All data are available and published online.

## Abbreviations

The following abbreviations are used in this manuscript:

TLM	third lower molar
PBMT	photobiomodulation therapy
M2M	mandibular second molars
AIN	alveolar inferior nerve
IL	interleukin
MCP	monocyte chemoattractant protein
TNF	tumor necrosis factor
EMI	electromagnetic interference
NF-kB	nuclear factor kappa B
LLLT	low-level laser therapy
